# Job-Related Performance and Quality of Life Benefits in First Responders Given Access to H-Wave^®^ Device Stimulation: A Retrospective Cohort Study

**DOI:** 10.3390/jpm12101674

**Published:** 2022-10-08

**Authors:** Tyler K. Williamson, Hugo C. Rodriguez, David Han, Stephen M. Norwood, Ashim Gupta

**Affiliations:** 1 University of the Incarnate Word School of Osteopathic Medicine, San Antonio, TX 78235, USA; 2 Department of Orthopaedic Surgery, Larkin Community Hospital, South Miami, FL 33143, USA; 3 Department of Management Science and Statistics, University of Texas at San Antonio, San Antonio, TX 78249, USA; 4 Retired Orthopaedic Surgeon, Austin, TX 78738, USA; 5 Future Biologics, Lawrenceville, GA 30043, USA; 6 Regenerative Orthopaedics, Noida 201301, UP, India

**Keywords:** H-Wave^®^, electrotherapy, neurostimulation, first responders, quality of life, job performance, range of motion, pain, chronic pain, pain reduction

## Abstract

Current chronic pain treatments primarily target symptoms and are often associated with harmful side-effects and complications, while safer non-invasive electrotherapies like H-Wave^®^ device stimulation (HWDS) have been less explored. The goal of this study is to evaluate first responder-reported effects of HWDS on job-related and quality-of-life measures. This is a retrospective cohort study where first responders were surveyed following voluntary use of HWDS regarding participant experience, frequency of use, job-related performance, and quality-of-life. Responses were analyzed using means comparison tests, while bivariate analysis assessed responses associated with HWDS usage. Overall, 92.9% of first responder HWDS users (26/28) reported a positive experience (*p* < 0.0001), with 82.1% citing pain reduction (*p* = 0.0013), while 78.6% indicated it would be beneficial to have future device access (*p* = 0.0046). Participants using H-Wave^®^ were at least six times more likely to report higher rates of benefit (100% vs. 0%, *p* = 0.022), including pain reduction (91.3% vs. 8.7%, *p* = 0.021) and improved range-of-motion (93.3% vs. 69.2%, *p* = 0.044). Spending more time with family was associated with better job performance following frequent HWDS use (50% vs. 8.3%, *p* = 0.032). Repetitive first responder H-Wave^®^ use, with minimal side effects and easy utilization, resulted in significant pain reduction, improvements in job performance and range-of-motion, and increased time spent with family, resulting in overall positive experiences and health benefits. Level of Evidence: III.

## 1. Introduction

Musculoskeletal pain, resulting from soft tissue injury and inflammation, continues to be a prevalent driver for primary care office visits, estimated to eventually occur in almost half of the adult population [1,2,3,4]. Such pain is often accompanied by functional deficits including decreased range of motion (ROM) and interference with activities of daily living (ADLs), often hampering work-related performance. With an increasing incidence of these conditions and related effects on quality of life (QoL), numerous and varied treatment strategies to address pain have been investigated and often overutilized. Several medications with differing mechanisms of action have been employed to counteract pain, including mainstay drugs acetaminophen and ibuprofen, with the latter better targeting both pain and underlying inflammation [5,6]. More pain-specific neuropathic modulators and opioids have often been added to alter pain pathways at multiple levels. Unfortunately, most available drugs only address the pain symptoms, having little effect on the underlying primary source stimulus. Due to multiple well-publicized side effects, particularly with addictive opioids, the concept of multimodal pain management has gained traction, incorporating concepts of physical exercise (with or without manual therapy), muscle and nerve stimulation, and even psychotherapy, while limiting drug prescription [7,8,9,10].

Electrotherapy, one category of alternative pain management, has the potential to target the nociception instigator. Commonly applied non-invasive electrotherapies like Transcutaneous Electrical Nerve Stimulation (TENS) and Neuromuscular Electrical Stimulation (NMES) have shown marginal effectiveness in pain reduction [11]. TENS provides some pain masking effects, while NMES induces tetanic fatiguing contractions, which limits rehabilitative benefits. Other more invasive techniques such as ultrasound guided percutaneous electrolysis have also shown some potential to reduce pain and improve functionality [12,13]. In contrast, H-Wave^®^ device stimulation (HWDS) triggers non-fatiguing, low-tension contractions that mimic voluntary muscle contraction [14,15]. Specifically, HWDS utilizes repeated stimulation of unique waveforms which: (1) activate muscle contraction, (2) enhance blood flow to tissues via nitric oxide–dependent vasodilation, (3) stimulate blood vessel formation (neovascularization), and (4) decrease or resolve edema [16,17]. Little to no side effects have been reported with device application for various diagnoses, even when used for sedentary- and frail-patient populations [18]. Previous HWDS studies have demonstrated that this relatively low-cost mobile modality, which typically requires only a single training session for effective use, represents a viable treatment option for neuropathic and musculoskeletal pain [18].

In addition to HWDS basic science and clinical principles, it is also very important to assess general usefulness, symptom resolution, and satisfaction from the perspective of device users. This investigation sought to broadly evaluate whether active first responders were likely to try the H-Wave^®^ device (when made available without cost) and to assess: (1) general efficacy across a variety of outcomes, (2) propensity to continue device use, and (3) interest in future device access. It was hypothesized that symptomatic first responders would report benefits from HWDS use, in terms of pain reduction and increased ROM, with an increased likelihood of supporting future workplace device availability.

## 2. Materials and Methods

### 2.1. Data Source and Study Design

This is a retrospective analysis of prospectively collected data from a survey of a cohort of first responder firefighters who agreed to voluntarily utilize HWDS for episodes of pain between March and May 2021. Volunteers received no special incentives or inducements. All participants provided informed consent and no protected health information was collected. This study was approved by the South Texas Orthopaedic Research Institute Institutional Review Board (study approval number: STORI08232022-1 and date of approval: 23 August 2022). General enrollment criteria for this dataset included being aged 18 or older with self-reported musculoskeletal or neuropathic pain. Participants were offered instruction on how to properly apply an H-Wave^®^ device at their station and then also offered access to a home device.

### 2.2. Data Collection

A survey was administered to qualifying first responders who elected to utilize an HWDS device. Survey participants were asked a pre-defined set of questions about their HWDS experiences (Appendix A Table A1), including effects on (1) pain levels, (2) sleep, (3) mood, (4) ROM, (5) missed work, (6) physical job performance, (7) time spent with family, (8) device use satisfaction, (9) need to file a workers’ compensation claim, (10) device-related adverse events, (11) number of times used (1–5, 6–10, 10+), (12) location of use (station, home), and (13) likelihood of future device use.

### 2.3. Statistical Analysis

The primary outcome was participant-reported experience following device use. Baseline demographic data and survey responses were compared using chi-squared and *t*-tests for categorical and continuous variables, respectively. Bivariate analysis assessed the association between survey responses related to use of HWDS. In all testing, significance was established a-priori for odds ratios and 95% confidence intervals (CI) exclusive of 1.0 and *p* < 0.05. All statistical analyses were conducted using SAS, version 9.4 (Cary, NC, USA).

## 3. Results

### 3.1. Cohort and Exclusion

**Figure 1:**
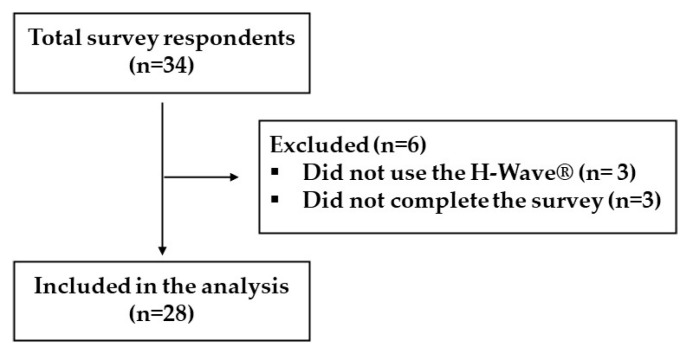
Inclusion flow diagram.

Of 34 total survey respondents, three participants did not use the H-Wave^®^ during the trial period and three failed to complete the survey(Excluded (*n* = 6)), resulting in an effective sample size of 28 (Figure 1). Of these 28 participants, 64.3% attended an H-Wave training session at their stations. No minor, moderate, or severe adverse effects were reported over the study duration with HWDS use.

### 3.2. Quality of Life-Related Survey Responses

The following QoL-related responses are recorded in Table 1. Overall, 92.9% of survey respondents stated they had a positive experience using H-Wave^®^ (*p* < 0.001). Functionality improved, with 82.1% of respondents stating that H-Wave^®^ treatment reduced pain (*p* = 0.001), while 53.6% reported increased ROM (*p* = 0.850) [not statistically significant]. Some 35.7% stated that H-Wave^®^ treatment improved sleep (*p* = 0.186), with 32.1% reporting improved mood (*p* = 0.089) and 32.1% also being able to spend more time with their family (*p* = 0.089) (not statistically significant).

### 3.3. Work-Related Survey Responses

The following work-related responses are recorded in Table 1. Of work-related experiences, 57.1% of survey respondents stated that H-Wave^®^ treatment improved their physical job performance (*p* = 0.571), while 35.7% reported it helped them to avoid missing work (*p* = 0.186) [both statistically insignificant]. Of only four survey participants responding to the question about workers’ compensation claims, three indicated H-Wave^®^ use had postponed or prevented filing a claim. These three also reported positive experiences, device use more than ten times, avoiding missed work, as well as spending more time with their family.

### 3.4. Bivariate Analysis

The bivariate analyses between survey questions are conveyed in Table 2. Of note, it was shown with statistical significance that participants who had attended H-Wave^®^ training at their station had higher rates of using the device ten or more times compared to those who never attended (72.2% vs. 20%, *p* = 0.016). Trained participants had much higher reported rates of pain reduction, while also indicating a positive experience using H-Wave^®^ (88.5% vs. 0%, *p* = 0.027). Participants who used H-Wave at least six times also reported much higher rates of benefit from treatment (100% vs. 0%, *p* = 0.022), including pain reduction (91.3% vs. 8.7%, *p* = 0.021) and increased ROM (93.3% vs. 69.2%, *p* = 0.044). Participants indicating mood improvement also had more time to spend with their family (66.7% vs. 15.9%, *p* = 0.025). Those spending more time with their family also reported better performance at their job (50% vs. 8.3%, *p* = 0.032). Participants reporting improvement of physical job performance were also more likely to state it would be beneficial to have future access to H-Wave^®^ (87.5% vs. 66.7%, *p* = 0.027).

## 4. Discussion

First responders assume unique and demanding responsibilities, admirably sacrificing themselves every day in the service to their communities, placing them at high risk of overexertion and chronic injuries. Previously published data has indicated that non-fatal musculoskeletal injuries, with associated pain and soreness, have been responsible for over half of the lost time at work by first responders [19,20,21]. As the average retirement age continues to rise, cumulative exposure and time-related degeneration will continue to be significant risk factors for workplace musculoskeletal injuries and episodic pain, further contributing to absenteeism [22,23]. First responders are among the most essential workers in our social structures, so it is critical that community efforts better focus on preventative and on-site care for their physical well-being, to maintain a consistent workforce and to protect these worthwhile and sustainable careers. This study demonstrates that repetitive H-Wave^®^ use, initiated at the workplace (station), resulted in self-reported pain reduction rates of 82%, with over half of surveyed first responders also reporting both improvement in job performance and ROM, leading to increased time spent with their family and an overall positive experience of 93%.

Treatment options for chronic musculoskeletal and neurological pain range widely, starting with simple observation/time and the rest-ice-compression-elevation (RICE) protocol, to pharmaceuticals, then physical therapy/chiropractor care, before invasive and surgical interventions are considered. The vast majority of musculoskeletal complaints, whether acute or chronic, can be treated effectively with nonoperative means. The recent consensus, considering the North American “opioid crisis”, strongly suggests that more efficacious treatments should follow a multimodal strategy, less dependent on controlled substances. Numerous studies seem to highlight interventions which more specifically target the primary pain source. Although opioids and numerous other treatment options may temporarily relieve symptoms, they provide no remedy to address the primary pain generator. NSAIDs, which target both pain symptoms and acute inflammation, can certainly be helpful in attenuating pain-intensifying moderators at the site of injury. Unfortunately, continuous NSAIDs use also has detrimental effects, including renal, gastric, and vascular compromise, that can appear as early as six weeks after treatment starts. In contrast, HWDS offers a non-fatiguing, drug-free, minimal-risk cascade of physiological and cellular mechanisms which targets and potentially eliminates the primary source of inflammation and pain generation, leading to restoration of function and QoL [18].

The findings of this study reinforce the positive conclusions of previous investigations which followed the repetitive use of HWDS, with no incidence of any adverse events. QoL measures were self-reported in first responders, and while HWDS often began alleviating some pain and dysfunction with initial treatment, their continued use seemed to be vital for maximum efficacy and health benefit [18]. Every participant using H-Wave at least 6-times self-reported some type of benefit, with over 90% of these first responders experiencing pain and ROM improvement.

First responders typically work 12- or 24-h shifts, often spending more time at work than at home, where sleep is a major necessity and time becomes misaligned with their family routines. Often this contributes to some stress and family dysfunction, resulting in an additional emotional burden for the first responder [24]. Functional and emotional improvement was reported with HWDS use, particularly in overall mood, which was significantly associated with more time spent with their family [25]. Research has also demonstrated that insufficient rest and sleep breaks greatly contributes to job fatigue amongst first responders, with work-life balance also playing a significant negative role [26]. More study participants reported sleep improvement when they also experienced H-Wave^®^ -related pain reduction (35.7% vs. 0%). Other potential downstream QoL benefits, beyond basic pain reduction and ROM restoration, seems likely and cannot be overstated, even if the present generalized survey study may not have fully captured other nuanced outcomes.

Increased functional improvements attributed to HWDS usage also translated into positive effects in the workplace. Those reporting that H-Wave^®^ allowed more time with their family also reported better job performance (50% vs. 8.3%). First responders were also more likely to state that it would be beneficial to have access to H-Wave^®^ again when also reporting improvements in job performance. Participants who attended H-Wave^®^ training at their station had much higher rates of subsequently using the device ten or more times compared to those who did not attend (72% vs. 20%). Only a single training session with an H-wave instructor was required for effective and more sustained utilization of HWDS [18,27]. Since first responders are constantly on the move, versatile mobile treatments can improve work-readiness, especially with modalities that function well in a variety of settings. The portable H-Wave^®^ device, with its flexible self-adhesive electrode placement, offers first responders a viable treatment option both on- and off-the-job, to effectively reduce pain and increase ROM, thereby improving work performance and sleep, resulting in more time spent with their family.

While these outcomes seem promising for first responders, there are certainly study limitations. A survey of only 28 participants without a control group limits analysis quality and increases potential confounding, volunteer, and sample bias. A lack of data regarding participant lifestyle, including factors like exercise, obesity, and smoking, could additionally confound results. The retrospective nature of the survey-based study additionally creates a risk for recall bias, with early positive experiences resulting in more prolonged use and better device familiarity.

This study demonstrated preferred continuation of H-Wave^®^ use after initial positive experiences in a cohort of first responders with musculoskeletal pain or injury. Considering future appropriate real-world application of these findings, future prospective studies are envisioned to analyze HWDS more critically in terms of causal effects. This will include condition-specific, prospective, multi-center, non-randomized, open-label, and randomized controlled double blinded trials to further establish general efficacy of H-Wave^®^. Further studies comparing efficacy of HWDS to conventional treatments, including exercise and manual therapy, dry or electrolysis needling, may be warranted to further define its efficacy profile. Possibly analyzing injured tissue pre- and post-HWDS via non-invasive techniques like ultrasound may additionally provide more objective insight into HWDS physiological healing benefits. Given a universal lack of access to H-Wave^®^ devices in similar work settings, as well as first responder organizational barriers, healthcare and policy efforts should begin to consider making H-Wave^®^ and other promising mobile treatments more readily available on-site for these most essential workers [18,20,28].

## 5. Conclusions

With a benign side-effect profile and relative ease of use, repetitive H-Wave^®^ treatment offers high rates of first responder-reported pain reduction, improvement in job performance and ROM, as well as increased time spent with family, leading to an overall positive experience and health benefit. Further retrospective studies with a significantly higher sample size and prospective, multi-center, open-label, non-randomized as well as double-blinded randomized controlled trials should be performed to add additional support for use of H-Wave^®^ as a key component of multi-modal non-opioid treatment of musculoskeletal and neuropathic pain; given its stellar safety profile and potential cost-savings compared to other questionable pain treatment options.

## Figures and Tables

**Table 1:** Proportion of Participants Reporting Benefits of H-Wave Device^®^ Stimulation.

Proportion of Survey Respondents	Number of Survey Respondents	Reported Benefit
92.86%	26	Positive experience *
82.14%	23	Less pain *
57.14%	16	Improved job performance
53.57%	15	Increased range of motion
35.71%	10	Improved sleep
35.71%	10	Avoided missed work
32.14%	9	Improved mood
32.14%	9	More time with family

* statistically significant.

**Table 2:** Bivariate contingency analysis of survey responses.

		**Avoid Missing Work**			**Total**
		**Yes**		**Not Sure**	
**Attended Training Session**	**Yes**	9		9	18
		32.14%		32.14%	64.29%
	**No**	1		9	10
		3.57%		32.14%	35.71%
**Total**		10		18	28
		35.71%		64.28%	100%
			
		**H-Wave^®^ Use Frequency**			**Total**
		**1 to 5**	**6 to 10**	**10+**	
**Attended Training Session**	**Yes**	1	4	13	18
		3.57%	14.29%	46.43%	64.29%
	**No**	4	4	2	10
		14.29%	14.29%	7.14%	35.71%
**Total**		5	8	15	28
		17.86%	28.57%	53.57%	100%
			
		**Reduction in Pain**			**Total**
		**Yes**		**No**	
**Benefit from H-Wave^®^**	**Yes**	23		3	26
		82.14%		10.71%	92.86%
	**No**	0		2	2
		0.00%		7.14%	7.14%
**Total**		23		5	28
		82.14%		17.86%	100%
			
		**Benefit from H-Wave^®^**			**Total**
		**Yes**		**No**	
**Use Frequency of H-Wave^®^**	**1 to 5**	3		2	5
		10.71%		7.14%	17.86%
	**6 to 10**	8		0	8
		28.57%		0.00%	28.57%
	**10+**	15		0	15
		53.57%		0.00%	53.57%
**Total**		26		2	28
		92.86%		7.14%	100%
			
		**Reduction in Pain**			**Total**
		**Yes**		**No**	
**Use Frequency of H-Wave^®^**	**1 to 5**	2		3	5
		7.14%		10.71%	17.86%
	**6 to 10**	8		0	8
		28.57%		0.00%	28.57%
	**10+**	13		2	15
		46.43%		7.14%	53.57%
**Total**		23		5	28
		82.14%		17.86%	100%
			
		**Increased Range-of-Motion (ROM)**			**Total**
		**Yes**		**No**	
**Use Frequency of H-Wave^®^**	**1 to 5**	1		4	5
		3.57%		14.29%	17.86%
	**6 to 10**	7		1	8
		25.00%		3.57%	28.57%
	**10+**	7		8	15
		25.00%		28.57%	53.57%
**Total**		15		13	28
		53.57%		46.43%	100%
			
		**Avoided Missing Work**			**Total**
		**Yes**		**No**	
**Use Frequency of H-Wave^®^**	**1 to 5**	0		0	5
		0.00%		0.00%	17.86%
	**6 to 10**	2		1	8
		7.14%		3.57%	28.57%
	**10+**	8		0	15
		28.57%		0.00%	53.57%
**Total**		10		1	28
		35.71%		3.57%	100%
			
		**Would Benefit from Further Use of H-Wave^®^**			**Total**
		**Yes**		**No**	
**Use Frequency of H-Wave^®^**	**1 to 5**	3		1	5
		10.71%		3.57%	17.86%
	**6 to 10**	5		3	8
		17.86%		10.71%	28.57%
	**10+**	14		0	15
		50.00%		0.00%	53.57%
**Total**		22		4	28
		78.57%		14.29%	100%
			
		**Improvement in Sleep**			**Total**
		**Yes**		**No**	
**Reduction of Pain**	**Yes**	10		13	23
		35.71%		46.43%	82.14%
	**No**	0		5	5
		0.00%		17.86%	17.86%
**Total**		10		18	28
		35.71%		64.29%	100%
			
		**Increased Range-of-Motion (ROM)**			**Total**
		**Yes**		**No**	
**Reduction of Pain**	**Yes**	15		8	23
		53.57%		28.57%	82.14%
	**No**	0		5	5
		0.00%		17.86%	17.86%
**Total**		15		13	28
		53.57%		46.43%	100%
			
		**Improvement in Physical Job Performance**			**Total**
		**Yes**		**No**	
**Reduction of Pain**	**Yes**	15		8	23
		53.57%		28.57%	82.14%
	**No**	1		4	5
		3.57%		14.29%	17.86%
**Total**		16		12	28
		57.14%		42.86%	100%
			
		**More Time Spent with Family**			**Total**
		**Yes**		**No**	
**Improvement in Mood**	**Yes**	6		3	9
		21.43%		10.71%	32.14%
	**No**	3		16	19
		10.71%		57.14%	67.86%
**Total**		9		19	28
		32.14%		67.86%	100%
			
		**More Time Spent with Family**			**Total**
		**Yes**		**No**	
**Increased Range of Motion**	**Yes**	7		8	15
		25.00%		28.57%	53.57%
	**No**	2		11	13
		7.14%		39.29%	46.43%
**Total**		9		19	28
		32.14%		67.86%	100%
			
		**More Time Spent with Family**			**Total**
		**Yes**		**No**	
**Improvement in Physical Job Performance**	**Yes**	8		8	16
		28.57%		28.57%	57.14%
	**No**	1		11	12
		3.57%		39.29%	42.86%
**Total**		9		19	28
		32.14%		67.86%	100%
			
		**Would Benefit from Further Use of H-Wave^®^**			**Total**
		**Yes**		**No**	
**Improvement in Physical Job Performance**	**Yes**	14		2	16
		50.00%		7.14%	57.14%
	**No**	8		4	12
		28.57%		14.29%	42.86%
**Total**		22		6	28
		78.57%		21.43%	100%

## Data Availability

Data is contained within the article.

## Appendix A

**Table A1:** Survey questions.

Topic	Questions
Attended Training Session	Did you attend an H-Wave training at your station?
Use of H-Wave^®^	Did you use the H-Wave?
Benefit From H-Wave^®^	Did you have a positive experience using H-Wave?
Reduction of Pain	Did your H-Wave treatment(s) reduce your pain?
Improvement in Sleep	Did your H-Wave treatment(s) improve your sleep?
Improvement in Mood	Did your H-Wave improve your mood?
Increased Range-of-Motion (ROM)	Did your H-Wave treatment(s) increase your range of motion?
Avoided Missing Work	Did your H-Wave treatment(s) helped you to avoid missed work?
Improvement in Physical Job Performance	Did your H-Wave treatment(s) improve physical job performance?
More Time Spent with Family	Did your H-Wave treatment(s) allow you to spend more time with family?
Postponed Workers Comp	Having access to H-Wave has postponed your need to file a work comp claim?
Use Frequency of H-Wave^®^	How many times did you use H-Wave? *Choose one of the following*: 1–5, 6–10, or 10+
Would Benefit from Further Use of H-Wave^®^	Would it be beneficial to you to have access to H-Wave again?
